# 基于非对称场流分离技术分离表征天麻多糖:空间位阻转变效应

**DOI:** 10.3724/SP.J.1123.2022.11020

**Published:** 2023-08-08

**Authors:** Mu WANG, Xirui ZHANG, Yuwei DOU, Hong YE, Haiyang DOU

**Affiliations:** 1.河北大学基础医学院,河北 保定 071000; 1. College of Basic Medical Sciences, Hebei University, Baoding 071000, China; 2.河北大学公共卫生学院,河北 保定 071000; 2. College of Public Health, Hebei University, Baoding 071000, China; 3.河北大学医学部,河北 保定 071000; 3. Health Science Center, Hebei University, Baoding 071000, China

**Keywords:** 非对称场流分离, 天麻多糖, 空间位阻转变, asymmetrical flow field-flow fractionation (AF4), *Gastrodia elata* polysaccharides (GEPs), steric transition

## Abstract

采用非对称场流分离(AF4)在线联用多角度光散射(MALS)检测器和示差折光(dRI)检测器(AF4-MALS-dRI)对天麻多糖(GEPs)的相对分子质量(*M*)、回转半径(*R*_g_)和构象进行了表征。GEPs粒径分布较宽,在AF4分离过程中存在空间位阻转变效应,导致粒径大小不同的GEPs分子被同时洗脱,无法准确表征其*M*、*R*_g_分布和构象。本文研究了恒定交叉流流速、指数衰减交叉流流速、样品质量浓度和垫片高度对空间位阻转变效应的影响。结果表明,较高的样品质量浓度会导致空间位阻转变现象的发生;垫片高度会影响样品的分离度和保留时间,改变空间位阻转变点(*d*_i_);对于多分散GEPs样品,指数衰减交叉流流速不仅能调整*d*_i_,而且能改善样品的分离度,缩短分析时间。在样品质量浓度为0.50 mg/mL、进样体积为50 μL、检测器流速为1.0 mL/min、垫片高度为350 μm、初始交叉流流速由0.2 mL/min呈指数衰减至0.05 mL/min、半衰期为2 min的条件下,解决了AF4分离GEPs时存在的空间位阻转变效应问题。此外,在最佳洗脱条件下研究了不同产地及不同超声时间对GEPs 的*M*、*R*_g_和构象的影响。结果表明,随着超声时间的增加,云南和四川产地GEPs的*R*_g_和*M*均减小,分布变窄;当超声时间为15 min时,云南产地GEPs为松散的高度支化构象,四川产地GEPs为球状构象;当超声时间增加到30 min或60 min时,两产地GEPs的构象主要为高度支化结构,表明GEPs发生了降解。实验结果证明,在最佳洗脱条件下,AF4-MALS-dRI对GEPs的表征具有良好的重复性,GEPs的*R*_g_和*M*相对标准偏差分别为0.5%和1.7%。

## Abstract (en)

Asymmetrical flow field-flow fractionation (AF4), a gentle tool for the separation and characterization of particles and macromolecules, has attracted increased interest in recent years owing to its broad dynamic size range and utilization of “open channel” voids in the packing or stationary phase. A steric transition phenomenon in which the sample elution mode change from the normal mode to the steric/hyperlayer mode occurs. Accurate characterization by AF4 requires the absence of steric transition, particularly when the sample has a broad size distribution, because the effect of the combination of different modes is difficult to interpret. In this study, the relative molecular mass (*M*), radius of gyration (*R*_g_), and conformation of *Gastrodia elata* polysaccharides (GEPs) were characterized using AF4 coupled with online multi-angle light scattering (MALS) and differential refractive index (dRI) detection (AF4-MALS-dRI). Steric transition was observed during GEP separation by AF4 owing to the broad size distribution of the molecules. This phenomenon would result in the inaccurate characterization of the GEPs in terms of *M* and *R*_g_ because two GEP groups of different sizes may elute together. In this study, the effects of constant and exponentially decaying cross-flow rates, sample mass concentration, and spacer thickness on steric transition were systematically investigated. The results indicated that a high GEP mass concentration (i. e., 0.75 mg/mL) can lead to steric transition. The spacer thickness affected the resolution and retention time of the GEPs and changed the steric transition point (*d*_i_). An exponentially decaying cross-flow rate not only adjusted the *d*_i_ of the polydisperse GEP samples but also improved the GEP resolution and shortened the analysis time. The influence of steric transition was solved under the following operating conditions: injected GEP mass concentration=0.5 mg/mL; injection volume=50 μL; spacer thickness=350 μm; detector flow rate=1.0 mL/min; and cross-flow rate exponentially decayed from 0.2 to 0.05 mL/min with a half-life of 2 min. Moreover, the influence of GEP origins and ultrasound treatment time on the *M* and *R*_g_ distributions and conformation of GEPs were investigated under the optimized operating conditions. The results showed that the *M* and *R*_g_ distributions of Yunnan and Sichuan GEPs decreased with increasing ultrasound time. When the ultrasound treatment time was 15 min, the Yunnan GEPs had a loosely hyperbranched chain conformation, whereas the Sichuan GEPs had a spherical conformation. When the ultrasound treatment time was increased to 30 or 60 min, the GEPs from both Yunnan and Sichuan had a hyperbranched chain conformation, indicating that ultrasound treatment resulted in GEP degradation. Under the same extraction conditions, GEPs from Yunnan had larger *M* and *R*_g_ values than those from Sichuan. AF4-MALS-dRI showed good repeatability for the characterization of GEPs under the optimized operating conditions. The relative standard deviations of *R*_g_ and *M* were 0.5% and 1.7%, respectively. The data presented in this study can be used as a starting point for in-depth studies on the structural bioactivity of GEPs.

天麻是我国传统的药食两源性中药材,以干燥块茎入药,有息风定惊、祛风通络的功效;作为食品,可用于人体日常的营养滋补。天麻含有80多种化合物,包括天麻素、甾醇、有机酸和多糖等,其中多糖在天麻中含量较高且具有重要的生物活性^[1,2]^。现代药理研究表明,天麻多糖(*Gastrodia elata* polysaccharides, GEPs)具有抗衰老、降血脂、抗肿瘤和保护神经等生物活性,已广泛应用于医药、食品和保健品领域^[3,4]^。研究证明,GEPs的生物活性与其相对分子质量(relative molecular mass, *M*)、回转半径(radius of gyration, *R*_g_)和构象有关^[5]^。因此,表征GEPs的*M*、*R*_g_及构象对进一步阐明其结构与功能之间的关系至关重要。

非对称场流分离(asymmetrical flow field-flow fractionation, AF4)是一种基于样品与外力场相互作用的分离技术。AF4分离流道中没有固定相,减少了多糖样品在分析过程中的剪切降解,使其具有较宽的尺寸检测范围(1 nm~50 μm)^[6]^。AF4在线联用多角度光散射(multi-angle light scattering, MALS)检测器和示差折光(differential refractive index, dRI)检测器(AF4-MALS-dRI)可以提供样品的*M*、*R*_g_及构象等信息^[7]^。AF4的洗脱模式包括正常模式、空间模式和超层模式。在正常模式下,根据AF4理论和Stokes-Einstein方程,将保留时间(*t*_r_)转化为流体动力学半径(*R*_h_)^[8]^,可以得到样品的粒径分布:


(1) $$R_{h}=\frac{kTV^{0}}{\pi \eta V_{c}w^{2}t^{0}}t_{r}$$

其中,*k*为玻尔兹曼常数,*T*为绝对温度(K), *V*^0^为空隙体积(mL),*η*为载液黏度(g/(cm·min)), *V*_c_为交叉流流速(mL/min), *w*为流道厚度(cm), *t*^0^为空隙时间(min)。样品粒径小于1 μm时为正常模式,在交叉流外力(*F*_F_)与自身布朗运动作用下达到平衡,由于粒径小的颗粒扩散系数大,平衡位置更接近于层流流速较快的流道中心,最终较小的粒子被优先洗脱;样品粒径大于1 μm时为空间模式,布朗运动可忽略不计,仅受向下的*F*_F_,使样品更靠近积累壁,此时粒径较大的颗粒更接近流道中心,被优先洗脱;在超层模式下,当样品在流体中运动产生的向上流体动力升力大于*F*_F_时,样品会在积累壁上端形成颗粒聚集层^[9,10]^,洗脱顺序与空间模式相同。当样品具有较宽的粒径分布时,AF4可能以正常模式和空间模式组合的形式分离,即空间位阻转变效应^[11]^。正常模式转换到空间模式时的粒径称为空间位阻转变点(*d*_i_),样品的粒径小于*d*_i_,以正常模式分离;样品的粒径大于*d*_i_,以空间/超层模式分离^[12]^。当样品的粒径分布包含*d*_i_时,在*d*_i_附近,粒径大和粒径小的样品成分被同时洗脱,无法实现分离,进而影响AF4表征*M*、*R*_g_分布及构象的准确性。前期研究表明,GEPs具有较宽的粒径分布,在AF4分离过程中可能存在空间位阻转变效应^[13]^。

本文以超声15 min提取的云南天麻多糖为研究对象,系统研究了AF4分离表征GEPs过程中恒定交叉流流速、指数衰减交叉流流速、样品质量浓度和垫片高度对*d*_i_的影响,在优化的条件下表征不同产地及不同超声时间提取GEPs的*M*、*R*_g_分布及构象。

## 1 实验部分

### 1.1 仪器、试剂与材料

Eclipse AF4场流分离系统(德国Wyatt公司); 1260 Infinity Ⅱ液相泵(美国Agilent公司); DAWN EOS多角度光散射检测器(美国Wyatt公司); RID-20A示差折光检测器(日本岛津公司)。SQP电子天平(北京赛多利斯科学仪器有限公司); MS-H550-Pro磁力搅拌器(北京大龙兴创实业有限公司); PE28 pH计(梅特勒-托利多(常州)精密仪器有限公司); RE-2000B旋转蒸发器(上海亚荣生化仪器厂); SCIENTZ-10N冷冻干燥机(宁波新芝生物科技股份有限公司); KQ5200DE数控超声波清洗机(昆山市超声仪器有限公司); TG16-WS离心机(湖南湘仪离心机仪器有限公司); UPR-Ⅱ-10T超纯水系统(西安优普仪器设备有限公司)。

云南天麻购自云南昭通老猫食品有限公司;四川天麻购自成都上本珍大药房有限公司;马脾铁蛋白购自美国Sigma-Aldrich公司;NaNO_3_和乙醇购自天津市科密欧化学试剂有限公司;丙酮购自天津市大茂化学试剂厂;NaOH和HCl购自上海麦克林生化科技有限公司。所用试剂均为分析级。

### 1.2 GEPs样品的制备

#### 1.2.1 天麻的处理

将四川和云南天麻洗净后低温烘干,用多功能粉碎机粉碎,过80目筛,得到天麻粉末。称取10.0 g天麻粉末,置于250 mL烧杯中,加入80 mL 80%乙醇水溶液,搅拌均匀,置于水浴锅中于60 ℃恒温浸泡2 h,重复操作2次后,以5000 r/min离心10 min,收集沉淀,晾干研磨后得到脱脂天麻粉末。

#### 1.2.2 GEPs的提取

参照文献^[13]^提取方法,具体如下:称取6.0 g脱脂天麻粉末,置于500 mL烧杯中,加入180 mL去离子水,于70 ℃、160 W条件下分别超声15、30、60 min后,以3000 r/min离心20 min,提取3次,合并上清液并浓缩,向浓缩液中加入5倍体积的95%乙醇水溶液,于4 ℃静置过夜;之后以5000 r/min离心10 min,收集沉淀,依次用95%乙醇水溶液和丙酮各洗涤3次后,再以5000 r/min离心10 min,收集沉淀,置于室温,待丙酮完全挥发后冷冻干燥得到不同超声时间下的云南GEPs (命名为Y-GEP-15、Y-GEP-30和Y-GEP-60)和四川GEPs (命名为S-GEP-15、S-GEP-30和S-GEP-60)。

#### 1.2.3 GEPs的溶解

分别称取2.0 mg不同产地的GEPs加入到20 mL样品瓶中,加入4.0 mL去离子水,在60 ℃水浴中以200 r/min搅拌2 h,将样品自然冷却至室温,得到质量浓度为0.50 mg/mL的GEPs溶液。

### 1.3 AF4分析天麻多糖

以Y-GEP-15溶液为例,进行AF4-MALS-dRI分析。按照1.2节方法配制Y-GEP-15溶液,采用350 μm聚酯垫片和10 kDa可再生纤维素膜组成AF4长流道,长度为26.5 cm;载液为5 mmol/L NaNO_3_去离子水溶液(pH 7);样品质量浓度为0.5 mg/mL,进样体积为50 μL,进样流速为0.2 mL/min,检测器流速为1.0 mL/min,初始交叉流流速从0.2 mL/min呈指数衰减至0.05 mL/min,半衰期(*t*_1/2_)为2 min。采用一阶Berry模式拟合测定*M*和*R*_g_^[14]^:


(2) $$\sqrt{\frac{Kc}{R_{\theta }}}=\sqrt{\frac{1}{M}+\frac{16\pi^{2}}{3\lambda^{2}}\times R_{g}^{2}\times {\mathrm{Sin}}^{2}\left(\frac{\theta }{2}\right)}$$

其中*K*为光学常数,*c*为样品质量浓度(g/mL),*R*_θ_为瑞利比,*λ*为波长(nm)。所获得的AF4数据用Astra 6.1.7软件进行处理,GEPs的折射率增量(*d*_n_/*d*_c_)为0.145 mL/g^[15]^。

## 2 结果与讨论

### 2.1 恒定交叉流流速对空间位阻转变效应的影响

AF4分离粒径分布较宽的样品时,可能出现空间位阻转变效应,影响AF4分离表征结果的准确性,因此可以通过改变恒定交叉流流速来调整*d*_i_,使样品成分的粒径均小于*d*_i_,样品全部在正常模式下洗脱^[12]^。以Y-GEP-15溶液为例,考察恒定交叉流流速对空间位阻转变的影响,将恒定交叉流流速分别设为0.2、0.3和0.5 mL/min,其他分析条件同1.3节。由图1可见,恒定交叉流流速为0.2 mL/min时,Y-GEP-15的*M*随*t*_r_的增加而增加,*M*分布起始阶段有凸起(3.1~7.5 min),表明可能存在空间位阻转变效应^[13]^;样品经0.45 μm针式过滤器(聚醚砜)过滤后,凸起消失,*M*随*t*_r_增加平滑上升,*M*分布为3.14×10^7^~2.40×10^9^, *R*_g_分布为69.7~368.7 nm。此外,过滤后样品的AF4-MALS-dRI洗脱峰在起始阶段(3.1~7.5 min)信号强度减弱,说明粒径较大的样品成分和粒径较小的样品成分被同时洗脱,证明在此分析条件下,Y-GEP-15样品存在空间位阻转变效应。有文献报道^[12]^,增加恒定交叉流流速,可以使*d*_i_向粒径小的方向移动。由图1可见,随着恒定交叉流流速增加,样品的AF4-MALS-dRI洗脱峰变宽并向长保留时间移动,这主要是由于增加恒定交叉流流速,样品平衡层更接近积累墙,此时的横向流速较小。恒定交叉流流速为0.5 mL/min时,AF4-MALS-dRI信号强度降低,这可能是由于样品与超滤膜表面发生了交联。

**图1:**
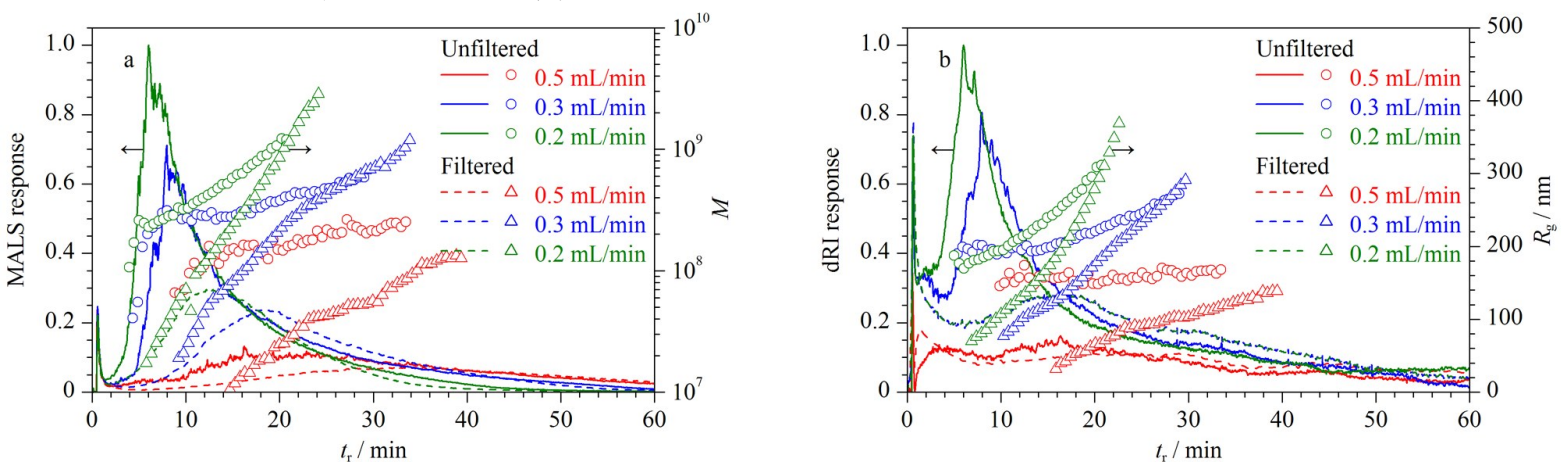
不同恒定交叉流流速下Y-GEP-15的AF4-MALS-dRI洗脱图谱、相对分子质量和回转半径分布图

### 2.2 指数衰减交叉流流速对空间位阻转变效应的影响

对于多分散样品,采用指数衰减交叉流流速不仅可以改善样品的分离度,而且可以缩短分析时间^[16]^。考察了指数衰减交叉流流速对空间位阻转变效应的影响,将初始交叉流流速分别设为0.2、0.3和0.5 mL/min,指数衰减至0.05 mL/min,*t*_1/2_为2 min,其他分析条件同1.3节。

由图2可见,当初始交叉流流速为0.2 mL/min、*t*_r_小于5.0 min时,过滤前后Y-GEP-15的AF4-MALS-dRI洗脱图谱、*M*和*R*_g_分布几乎相同,过滤前Y-GEP-15的*M*分布为3.71×10^7^~4.29×10^8^, *R*_g_分布为93.1~190.3 nm,且随*t*_r_的增加而平滑上升,说明初始交叉流流速为0.2 mL/min时能有效解决空间位阻转变效应问题;*t*_r_大于5.0 min、过滤后样品的AF4-MALS-dRI洗脱峰信号强度减弱,这是由于过滤掉了部分粒径大的样品。随着初始交叉流流速增加到0.3 mL/min或0.5 mL/min,*t*_r_小于7.0 min、过滤后样品的AF4-MALS-dRI洗脱峰信号强度降低,说明一些粒径较大的成分被优先洗脱,存在空间位阻转变效应。与恒定交叉流流速相比(图1),指数衰减交叉流流速缩短了分析时间。综上,选择初始交叉流流速为0.2 mL/min,指数衰减至0.05 mL/min,*t*_1/2_为2 min。

**图2:**
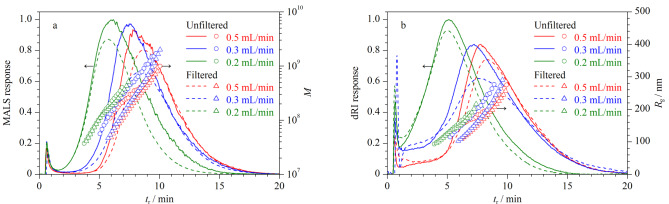
指数衰减交叉流不同初始流速下Y-GEP-15的AF4-MALS-dRI洗脱图谱、*M*和*R*_g_分布图

### 2.3 样品质量浓度对空间位阻转变效应的影响

样品质量浓度是影响分离度的一个重要因素。样品质量浓度较低时,洗脱峰信号强度较弱;反之,过量的样品会与超滤膜发生交联^[17]^。考察了样品质量浓度对空间位阻转变效应的影响,采用质量浓度分别为0.25、0.50和0.75 mg/mL的Y-GEP-15样品,其他分析条件同1.3节。结果如图3所示,样品质量浓度为0.25 mg/mL时,AF4-MALS-dRI洗脱峰信号较弱,无空间位阻转变效应;随着样品质量浓度增加至0.75 mg/mL, AF4-MALS-dRI洗脱峰信号增强,*t*_r_减少,分离度逐渐变差,出现空间位阻转变效应。这是由于流道中样品过载,在聚集过程中样品质量浓度较高,部分样品无法达到稳态弛豫位置,导致一些粒径较大的样品被优先洗脱^[18]^。样品质量浓度为0.50 mg/mL时,样品峰对称性良好,过滤前后的*t*_r_一致,*R*_g_和*M*分布趋势呈线性。综上,选择样品质量浓度为0.50 mg/mL。

**图3:**
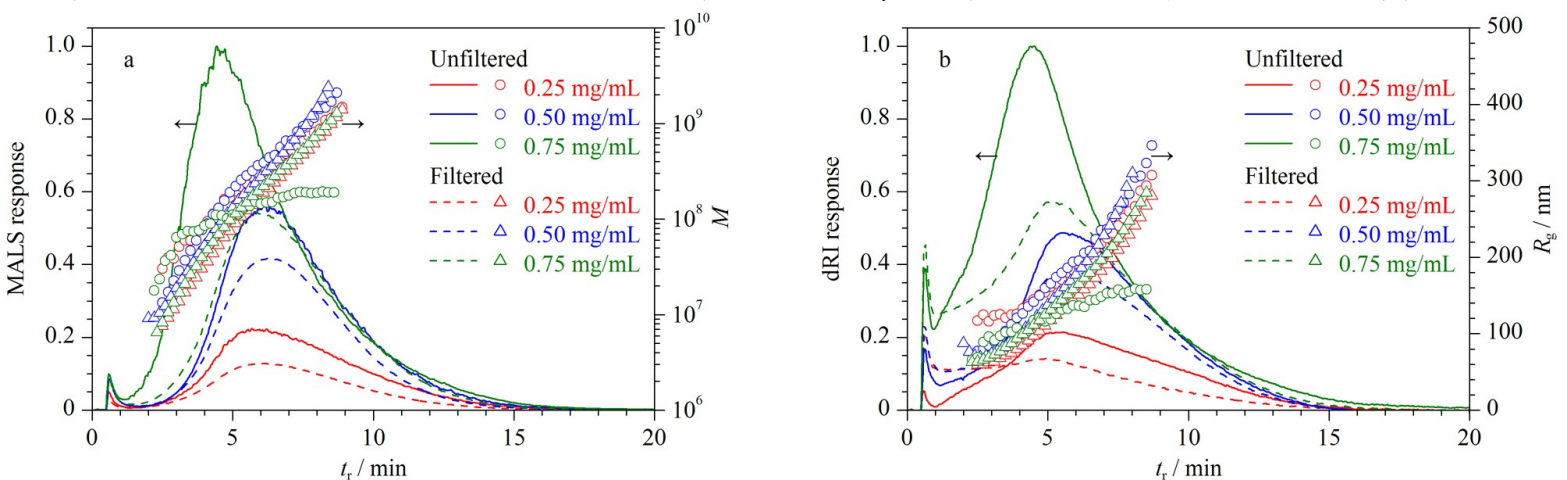
不同质量浓度下Y-GEP-15的AF4-MALS-dRI洗脱图谱、*M*和*R*_g_分布图

### 2.4 垫片高度对空间位阻转变效应的影响

垫片高度决定AF4流道高度,流道高度的改变会使保留率(*R*=*t*^0^/*t*_r_)发生变化,进而改变*d*_i_^[19]^。考察了垫片高度对空间位阻转变效应的影响,采用高度分别为250、350和490 μm的聚酯垫片,其他分析条件同1.3节。由图4可见,垫片高度为250 μm时,在起始阶段(0.8~2.2 min),过滤后Y-GEP-15的*M*和*R*_g_变小,说明粒径较大的样品成分被优先洗脱,存在空间位阻转变效应。随着垫片高度的增加,样品峰分离度变好,洗脱峰信号强度减弱,*t*_r_增加,空间位阻转变效应消失^[11]^,这是由于垫片高度增加时,*R*减小,Y-GEP-15样品的*d*_i_发生改变,所受到的层流流速越小,洗脱时间越长^[12]^。垫片高度为350 μm和490 μm时,Y-GEP-15均不存在空间位阻转变效应;垫片高度为350 μm时,Y-GEP-15的样品洗脱峰信号更强,对称性良好,分析时间短。综上,选择垫片高度为350 μm。

**图4:**
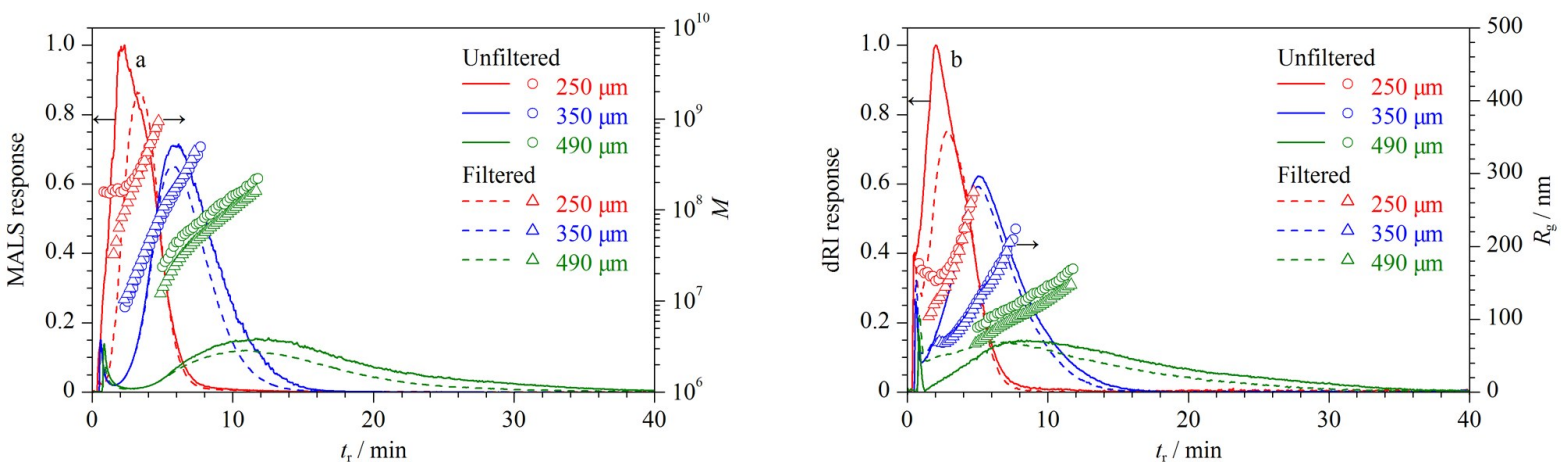
不同垫片高度下Y-GEP-15的AF4-MALS-dRI洗脱图谱、*M*和*R*_g_分布图

### 2.5 AF4分析天麻多糖方法的重复性

为了考察AF4-MALS-dRI分离表征GEPs的可行性,在1.3节条件下,对Y-GEP-15样品进行分析,重复3次。结果如图5所示,3次进样的MALS和dRI信号趋势及强度基本一致,*R*_g_相对标准偏差为0.5%, *M*相对标准偏差为1.7%,说明AF4-MALS-dRI分离表征GEPs的重复性良好。

**图5:**
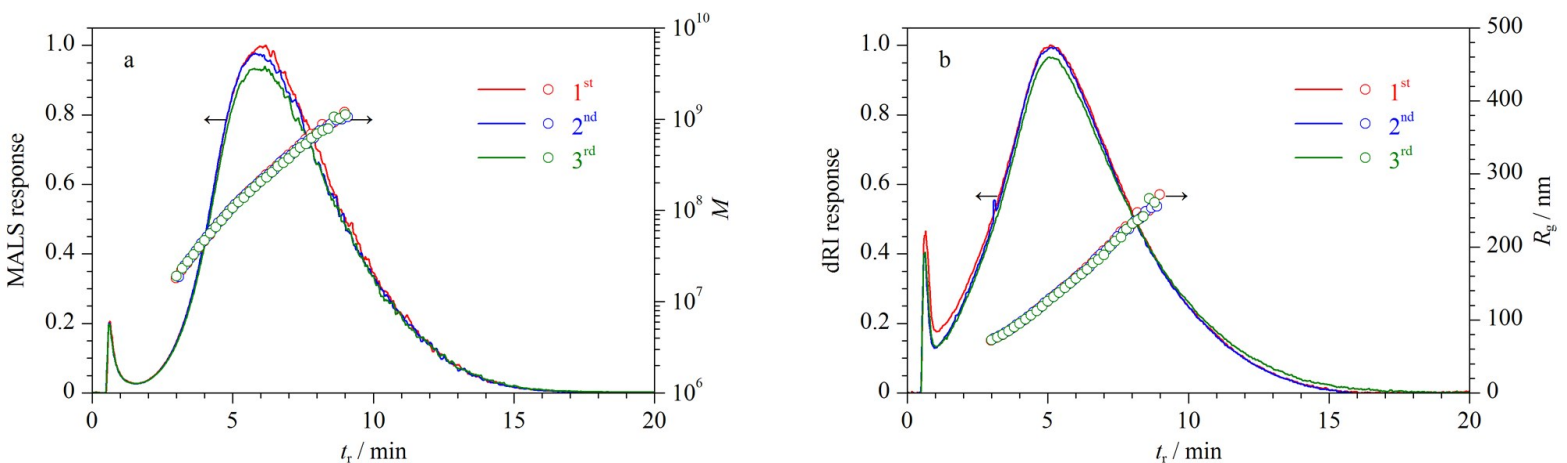
Y-GEP-15的AF4-MALS-dRI洗脱图谱、*M*和*R*_g_分布图的重复性

### 2.6 不同产地天麻多糖的AF4-MALS-dRI分析

在1.3节条件下,分析不同产地及不同超声时间提取的GEPs。结果如图6所示,随着超声时间的增加,不同产地GEPs的AF4-dRI洗脱峰信号增强,AF4-MALS洗脱峰信号减弱,*R*_g_和*M*均减小,分布变窄,说明超声辅助热水法提取GEPs时,超声处理时间越长,GEPs粒径越小。这可能是由于超声波的机械、空化和热效应能够破碎天麻的细胞壁,加快内部多糖溶出,同时导致GEPs降解^[20]^。

**图6:**
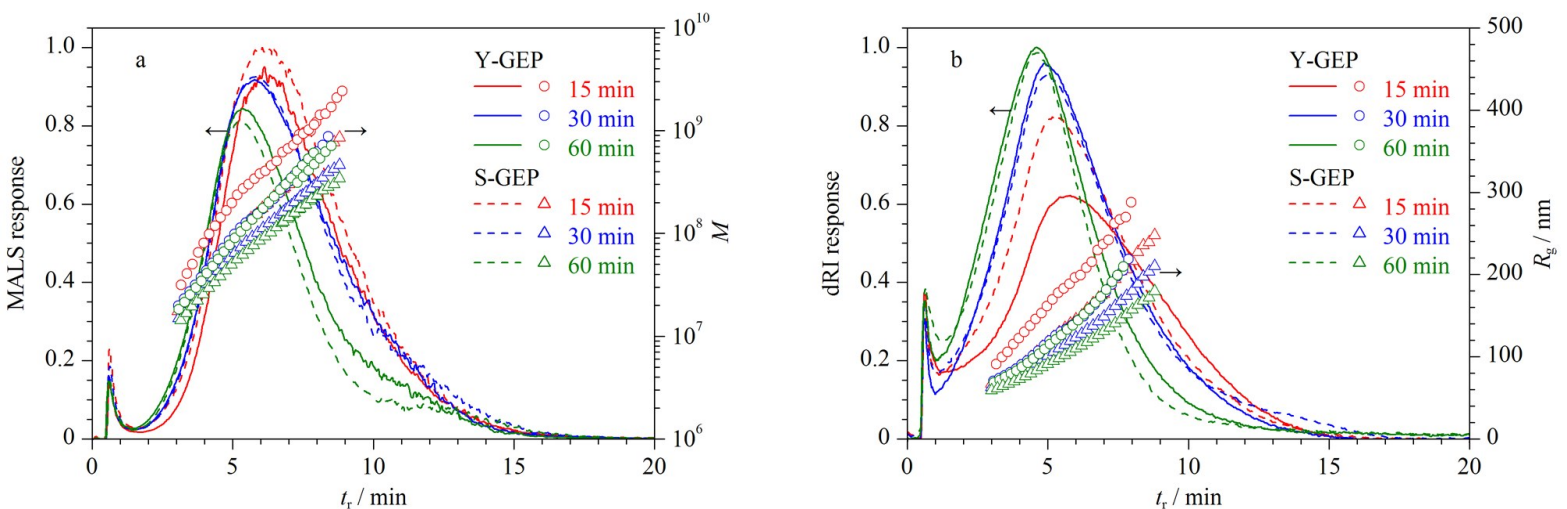
不同产地及不同超声时间提取GEPs的AF4-MALS-dRI洗脱图谱、*M*和*R*_g_分布图

不同产地及不同超声时间提取GEPs的分子特性数据(*M*、*R*_g_和聚合物分散指数(PDI))见表1。由表1可见,不同产地GEPs的PDI均大于1,说明GEPs为多分散性多糖。随着超声时间的增加,PDI、*M*和*R*_g_的分布范围均减小,说明超声作用降解了GEPs,使其趋于均一性多糖。不同产地GEPs的PDI、*M*和*R*_g_分布存在差异,在同一提取条件下,云南产地GEPs的PDI、*M*和*R*_g_分布均高于四川产地。

**表1:** 不同产地及不同超声时间提取GEPs的分子特性

Sample	Habitat	Ultrasound time/min	*M* distribution	*R*_g_ distribution/nm	PDI/(*M*_w_/*M*_n_)
Y-GEP-15	Yunnan	15	3.14×10^7^-2.40×10^9^	90.9-287.7	2.93
Y-GEP-30	Yunnan	30	1.98×10^7^-8.70×10^8^	87.1-234.2	2.91
Y-GEP-60	Yunnan	60	1.82×10^7^-7.17×10^8^	68.5-209.9	2.83
S-GEP-15	Sichuan	15	1.75×10^7^-8.58×10^8^	67.2-247.3	2.37
S-GEP-30	Sichuan	30	1.49×10^7^-4.46×10^8^	62.7-210.0	1.79
S-GEP-60	Sichuan	60	1.42×10^7^-3.44×10^8^	58.8-209.9	1.61

Y-GEP-30 and Y-GEP-60: Yunnan GEPs treated with ultrasound times of 30 min and 60 min; S-GEP-15, S-GEP-30 and S-GEP-60: Sichuan GEPs treated with ultrasound times of 15, 30 and 60 min. PDI: polymer dispersity index; *M*_w_: weight-average molecular weight; *M*_n_: number-average molecular weight.

AF4不仅是一种温和的分离技术,而且可以对样品的*R*_h_进行表征(公式1)。*R*_g_/*R*_h_可以用于评估样品的构象,*R*_g_/*R*_h_<0.7为高度分支/膨胀结构,*R*_g_/*R*_h_=0.778为实心球结构,*R*_g_/*R*_h_介于1.0~1.5为多分支结构^[21,22]^。采用AF4-MALS-dRI对不同产地及不同超声时间提取GEPs的*R*_g_/*R*_h_进行表征。结果如图7a所示,相对分子质量为10^7^~10^9^时,Y-GEP-15的*R*_g_/*R*_h_介于0.8~1.1, Y-GEP-30和Y-GEP-60的*R*_g_/*R*_h_介于0.6~0.8, S-GEP-15的*R*_g_/*R*_h_介于0.65~0.78, S-GEP-30和S-GEP-60的*R*_g_/*R*_h_介于0.58~0.66,说明GEPs样品主要是高度支化结构。此外,标度指数(*v*)常用于评估样品的构象^[23]^, *v*=1为棒状结构,*v*=0.5~0.6为无规线团结构,*v*=0.3为实心球结构,*v*=0.28~0.35为高度支化结构^[24,25]^。由图7b可知,Y-GEP-15、Y-GEP-30、Y-GEP-60、S-GEP-15、S-GEP-30、S-GEP-60的*v*分别为0.324、0.341、0.339、0.341、0.366、0.350,说明GEPs主要为高度分支结构,与*R*_g_/*R*_h_表征结果一致。

**图7:**
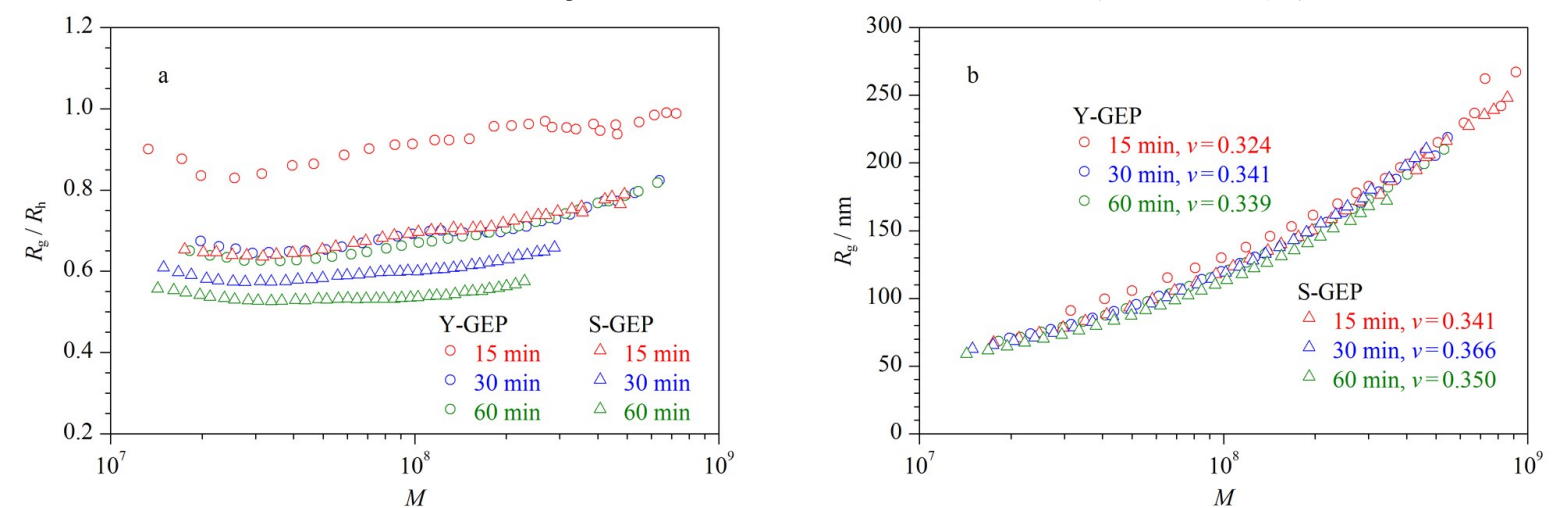
不同产地及不同超声时间提取GEPs的构象

## 3 结论

本研究采用超声辅助热水提取法在不同超声时间下提取了四川和云南产地的GEPs;采用AF4-MALS-dRI研究了不同洗脱条件对空间位阻转变效应的影响。研究结果表明,对于粒径分布宽的GEPs,恒定交叉流流速、指数衰减交叉流流速、样品质量浓度和垫片高度均影响空间位阻转变效应。通过使用指数衰减交叉流流速,改变垫片高度来调整*d*_i_,将处于空间反转范围的样品转换成正常洗脱模式分离,解决了GEPs分离过程中的空间位阻转变效应问题,改善了样品的分离度,缩短了分析时间。结果证明在优化的洗脱条件下,AF4-MALS-dRI是分离表征GEPs的有力工具,可为今后解析GEPs的构效关系提供支持。
